# COVID-19 Response in Sub-Saharan Low-Resource Setting: Healthcare Soldiers Need Bullets

**DOI:** 10.4269/ajtmh.20-0543

**Published:** 2020-06-22

**Authors:** Denis Mukwege, Guy-Bernard Cadière, Olivier Vandenberg

**Affiliations:** 1 Gynaecology and General Surgery, Panzi General Referral Hospital, Bukavu, Democratic Republic of Congo;; 2 Faculty of Medicine, Evangelical University in Africa, Bukavu, Democratic Republic of Congo;; 3 Department of Gastrointestinal Surgery, European School of Laparoscopic Surgery, Saint-Pierre University Hospital, Université Libre de Bruxelles, Brussels, Belgium;; 4 Centre for Environmental Health and Occupational Health, School of Public Health, Université Libre de Bruxelles (ULB), Brussels, Belgium;; 5 Innovation and Business Development Unit, LHUB - ULB, Université Libre de Bruxelles, Brussels, Belgium;; 6 Division of Infection and Immunity, Faculty of Medical Sciences, University College London, London, United Kingdom

At the time of this writing (May 27, 2020), there are few people infected with COVID-19 in my country, the Democratic Republic of the Congo (DRC), compared with the rest of the world. On May 22nd, a cumulative total of 1,731 confirmed COVID-19 cases with 60 deaths were reported by the WHO. Among them, only four cases were identified by national authorities in the province of South-Kivu. Several assumptions have been made about this low rate, the most likely of which is that the epidemic has not yet spread in my region. Prevention remains the key element in the fight against COVID-19 before spreading.

However, implementing strict containment of the whole population as recommended in industrialized countries is not feasible in my setting because most people have to go out for food every day. Given this, a prevention strategy based on the rapid identification of infected patients and their contacts with subsequent isolation is crucial. Often considered as difficult to implement in a resource-limited setting, our country previously successfully implemented such strategy during the last Ebola outbreak in 2019.

Since March 26, 2020, and far before the identification of the first COVID-19 case in this eastern part of the DRC, I have been commissioned to lead the health commission for response to the COVID-19 pandemic in the province of South-Kivu. Because I firmly think it is the cornerstone to block the spreading of COVID-19 in our setting, the first recommendation I messaged to the public was the reinforcement of standard infection control measures including systematic handwashing, and coughing in the elbow. In addition, thanks to the support of national and international advisory boards, we implemented strong public health containment measures comprising wearing of masks in public places, strict social distancing, placing people older than 60 years in isolation, and the curfew of Bukavu (the capital of the South-Kivu Province), and the closure of all provincial borders, churches, and educational facilities has been made mandatory. Last but not least, we put in place at the same period the rapid identification, isolation, and care of infected individuals and the monitoring of their contacts.

Even though it is difficult, we can take on this strategy with our own resources with the exception of the required mass use of SARS-COV-2 molecular diagnostic tests. These tests are currently not available in our province or centralized at the national level, leading to unacceptable delays in diagnosis and rendering containment measures hardly acceptable to the population. Indeed, we are waiting for more than 10 days before getting laboratory results from the national reference laboratory. As a result, we are witnessing today within the population a questioning of the COVID-19 threat and a resumption of regional economic exchanges, making it difficult to sustain proposed prevention measures. Because we fail to rapidly confirm the infection, suspected COVID-19 patients who are isolated pending the microbiological confirmation feel quarantine as a subject condemned to prison for witchcraft. Furthermore, our inability reopens the doors of all assumptions, including the discrimination between individuals and the feeling of being labeled COVID-19 without any evidence.

Regarding the risk of SARS-COV-2 spreading into the population, we have developed a local protocol that deviates from the current international guidelines restricting the use of recently developed COVID-19 rapid diagnostic test (RDT) to research settings pending the results of ongoing evaluation. In our province, we have implemented the use of COVID-19 RDT in an integrative diagnostic strategy combining SARS-CoV-2 antigen detection, molecular point-of-care (POCT), and the molecular diagnostics performed at the national level on large automated platforms.

In our algorithm, we start with a rapid COVID-19 antigen test for COVID-19–suspected people suffering of cough and fever. We acknowledge that the COVID-19 Ag Respi-Strip (Coris BioConcept, Gembloux, Belgium) tests we use have a sensitivity that is too low (overall 60%, reaching 85% for samples with high viral load) to be used as a single diagnostic test at triage; therefore, we send all negative samples to the reference laboratory for qRT-PCR testing in the frame of the national large screening strategy. Because the latter often require a turnaround time of more than 10 days, we are considering using other molecular POCT on patients suffering from severe diseases and requiring hospitalization.

Because such RDTs can be operated by trained laboratory technicians and aware of the limitations of the antigenic RDT, our decision algorithm has the advantages to expand decentralized testing in district medical laboratories in a period where rapid identification of COVID-19–infected people is crucial to prevent community transmission. Even with this approach, we catch only one of two infected patients with such RDT.

As feared, we now are seeing the opposite of Europe; the numbers of people infected by COVID-19 are now increasing exponentially, especially in the last 3–4 days. Indeed, since May 16th, date of the launch of our diagnostic algorithm, we are currently bracing for the worst, as nine additional cases of COVID-19 infection have been found in Bukavu. Among them, six healthcare workers have been infected, leading us to segregate teams caring for suspect and confirmed cases of COVID-19 from those managing other patients. In a context of shortage of personal protective gear, we hope this will minimize the risk of cross infection of patients and healthcare workers.

However, our control and prevention strategy will prove to be in vain if we do not get quickly molecular diagnostic tests and protective equipment. Indeed because of the inability of national health authorities and regional partners (e.g., African CDC and UNICEF) to provide such equipment, we are still waiting for the holy grail of diagnosis recommended by international guidelines.

Such supply is for us the only way to help us flatten the curve and contain the pandemic, which in a local context of fragile peace may fuel insecurity and social unrest.

